# Born Toon Soon: Preterm birth matters

**DOI:** 10.1186/1742-4755-10-S1-S1

**Published:** 2013-11-15

**Authors:** Christopher P Howson, Mary V Kinney, Lori McDougall, Joy E Lawn

**Affiliations:** 1 March of Dimes, White Plains, NY, USA; 2 Saving Newborn Lives/Save the Children, Cape Town, South Africa; 3 The Partnership for Maternal Newborn and Child Health, Geneva, Switzerland; 4 MARCH, London School of Hygiene & Tropical Medicine, UK; 5 Saving Newborn Lives/Save the Children

## Abstract

Urgent action is needed to address preterm birth given that the first country-level estimates show that globally 15 million babies are born too soon and rates are increasing in most countries with reliable time trend data. As the first in a supplement entitled "Born Too Soon", this paper focuses on the global policy context. Preterm birth is critical for progress on Millennium Development Goal 4 (MDG) for child survival by 2015 and beyond, and gives added value to maternal health (MDG 5) investments also linking to non-communicable diseases. For preterm babies who survive, the additional burden of prematurity-related disability may affect families and health systems. Prematurity is an explicit priority in many high-income settings; however, more attention is needed especially in low- and middle-income countries where the invisibility of preterm birth as well as its myths and misconceptions have slowed action on prevention and care. Recent global attention to preterm birth hit a tipping point in 2012, with the May 2 publication of *Born Too Soon: The Global Action Report on Preterm Birth *and with the 2^nd ^annual World Prematurity Day on November 17 which mobilised the actions of partners in many countries to address preterm birth and newborn health. Interventions to strengthen preterm birth prevention and care span the continuum of care for reproductive, maternal, newborn and child health. Both prevention of preterm birth and implementation of care of premature babies require more research, as well as more policy attention and programmatic investment.

**Declaration:**

This article is part of a supplement jointly funded by Save the Children's Saving Newborn Lives programme through a grant from The Bill & Melinda Gates Foundation and March of Dimes Foundation and published in collaboration with the World Health Organization (WHO). The original article was published in PDF format in the WHO Report "Born Too Soon: the global action report on preterm birth (ISBN 978 92 4 150343 30). The article has been reformatted for journal publication and has undergone peer review according to *Reproductive Health*'s standard process for supplements and may feature some variations in content when compared to the original report. This co-publication makes the article available to the community in a full-text format.

## Preterm birth matters in every country

More than one in 10 of the world's babies born in 2010 were born preterm (defined as before 37 weeks of gestation), resulting in an estimated 14.9 million preterm births [1]. Of these, more than one million died as a direct result of their prematurity [2]. Being born moderately preterm with or without fetal growth restriction acted as a risk factor for a further one million neonatal deaths from causes such as infections [3]. Prematurity is now the second leading cause of death in children under-5 years and the single most important direct cause of death in the critical first month of life [2]. For the babies who survive, many face a lifetime of significant disability [3]. Preterm birth accounts for 3.1% of all Disability Adjusted Life Years (DALYs) in the Global Burden of Disease, more than for HIV and malaria [4]. Given the frequency of preterm birth worldwide, it is likely that most people will experience the tragedy of preterm birth at some point in their lives, either in family members or indirectly through friends.

Prematurity is an explicit public health priority in many high-income countries. However, until recently, the lack of data on preterm birth at the country level has rendered preterm birth almost invisible and hampered action in response in most low- and middle-income countries (World Bank. (2012). How we classify countries. Retrieved March 27, 2012 from: http://data.worldbank.org/about/country-classifications). In May 2012, more than 100 experts representing almost 50 agencies, universities, organization and parent groups came together to produce *Born Too Soon: The Global Action Report on Preterm Birth *[5]. The report featured the first ever country-level estimates on preterm birth prevalence developed by the Child Health Epidemiology Reference Group, The London School of Hygiene & Tropical Medicine and WHO and published in *The Lancet *[1]. These estimates show that prematurity rates are increasing in almost all countries with reliable time trend data [1].

The implications of being born too soon extend beyond the neonatal period throughout the life cycle. Babies who are born before they are physically ready to face the world often require special care and face greater risks of serious health problems, including cerebral palsy, intellectual impairment, chronic lung disease and vision and hearing loss. This added dimension of lifelong disability exacts a high toll on individuals born preterm, their families and the communities in which they live [6].

There are two-way linkages between preterm birth, low birthweight and non-communicable diseases (NCDs) such as diabetes and hypertension. Firstly, women with these NCDs have an elevated risk of having a low birthweight baby due to prematurity or other causes, demanding increased attention to maternal health and care, including the antenatal diagnosis and management of NCDs [7]. Premature babies, in turn, are at greater risk of developing NCDs like hypertension and diabetes later in life and, if female, of having a preterm and/or low birthweight baby herself. Thus, prematurity not only affects a newborn directly but can also result in a vicious intergenerational cycle of risk [8]. The link between prematurity and an increased risk of hypertension, diabetes and other NCDs takes on an added public health importance when the reported increases in the rates of NCDs worldwide are taken into consideration. Currently, nine million people under the age of 60 years die from NCDs per year, accounting for more than 63% of all deaths, with the greatest burden in Africa and other low- income regions where preterm birth rates are also high [9]. With pregestational diabetes and hypertension reported to increase the risk of having a preterm delivery in the US by 38% and 33%, respectively, it is clear that the problem of preterm birth should be a major concern to policy-makers, donor organisations and other stakeholders in the NCD as well as RMNCH communities [10].

## The Millennium Development Goals and beyond

The substantial decline in maternal, newborn and child deaths in high-income countries in the early and middle 20th century was a public health triumph. Much of this decline was due to improvements in socioeconomic, sanitation and educational conditions and in population health, most notably a reduction in malnutrition and infectious diseases [11,12]. These advances in public health also resulted from strengthened political will prompted by public pressure, often by health professionals who demanded attention to and investment in necessary sanitary measures, drugs and technologies [13]. Many low- and middle-income countries are now experiencing a similar "health transition," defined as an "encompassing relationship among demographic, epidemiologic and health changes that collectively and independently have an impact on the health of a population, the financing of health care and the development of health systems" [14].

Recent acceleration in mortality reduction for mothers and for children aged between 1 and 59 months has been driven, in part, by the Millennium Development Goal (MDG) framework [15,16]. Established by 189 member states in 2000 with a target date of 2015 [17], the eight interlinking global goals provide benchmarks by which to measure success [18]. As such, they have mobilised common action to accelerate progress for the world's poorest families. These goals put reproductive, maternal, newborn and child health (RMNCH) on the global stage by raising their visibility politically and socially and have helped unite the development community in a common framework for action. The need to monitor progress has also led to improved and more frequent use of health metrics and to collaboration and consensus on how to strengthen primary health care systems, from community-based interventions to the first referral-level facility at which emergency obstetric care is available [19].

**Figure 1:**
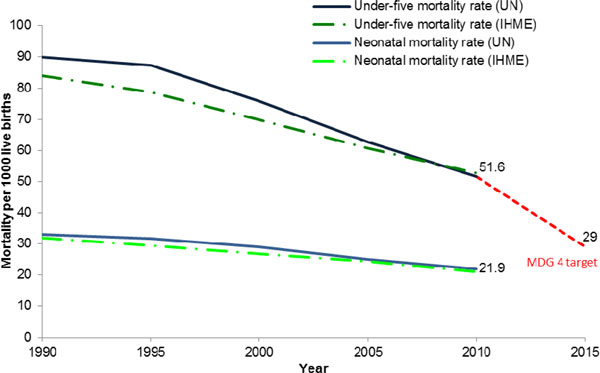
**Millennium Development Goal 4 Progress**. Source: Adapted from Lawn et al., 2012. Data from UN Interagency Group for Child Mortality Estimates (UNICEF, 2013) and the Institute for Health Metrics and Evaluation (Lozano et al., 2011). Note: MDG 4 target reflects a 2/3 reduction from the under-5 mortality rate in 1990.

MDG 4 calls for a reduction in the under-5 mortality rate by two-thirds between 1990 and 2015. MDG 5 has two targets: the first calling for a reduction in the maternal mortality ratio by three-quarters and the second for universal access to reproductive health during the same period. Even with the increased visibility and progress that MDGs 4 and 5 have brought to maternal and child survival, the rate of decline for mortality reduction remains insufficient to reach the targets, particularly in sub-Saharan Africa and South Asia (Figure 1). For example, only 37 countries (out of around 180) are currently on track to achieve the MDG 4 target in 2015, although another 26 are close to the target [15]. One important barrier to progress has been the failure to reduce neonatal deaths and particularly those due to prematurity [20]. Child survival programmes have primarily focused on important causes of death after the first four weeks of life such as pneumonia, diarrhoea, malaria and vaccine-preventable conditions [21], resulting in a significant decline in under-5 mortality rates. While important, the concomitant lack of attention to important causes of neonatal mortality like preterm birth, which is now the single largest cause of neonatal mortality accounting directly for one-third of neonatal deaths, has resulted in neonatal deaths becoming an increasing proportion of under-5 deaths, from 37% in 1990 to 44% in 2012 [22], and demonstrating a slower rate of decline than that for under-5 deaths (Figure 1) [23,24].

**Figure 2:**
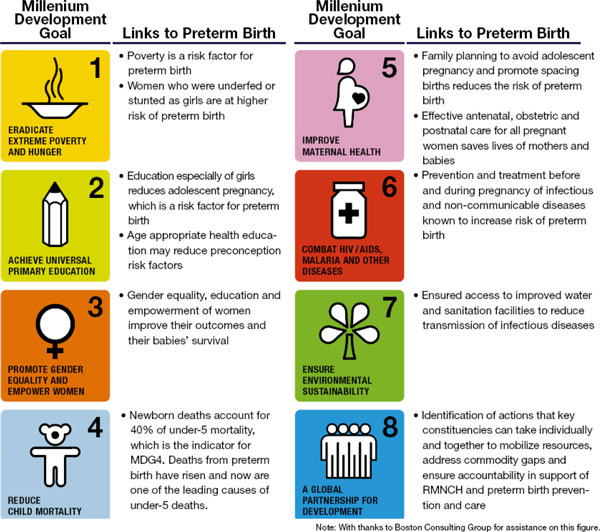
**How the Millennium Development Goals Link to Prevention and Care of Preterm Births**. Source: Born Too Soon report [5].

**Figure 3:**
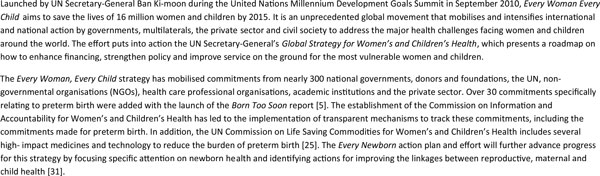
***Every Woman, Every Child *and now *Every Newborn***. From Born Too Soon report [5].

The actions presented in *Born Too Soon *[25], if implemented quickly, will accelerate the reduction of neonatal deaths in the last critical days to the 2015 target and beyond. In addition, they will contribute to improved maternal health and care, thus benefiting women directly. However, when considered in the full context of public health and development, these actions are importantly linked to all eight MDGs (Figure 2) and should not be thought of as an isolated program of "prematurity care and prevention." The actions require the engagement of organisations and expertise, not only from across the RMNCH spectrum, but also from non-health sectors such as education and environmental sustainability. In addition, they must be firmly embedded within existing frameworks for action and accountability, most notably the *Every Woman, Every Child *effort led by UN Secretary-General Ban Ki-moon (Figure 3). Such engagement will serve to accelerate progress towards all eight MDGs and have an effect beyond improving maternal, newborn and child survival.

## Preterm birth matters as a public health problem

Preterm births have been accorded a high public health priority in high-income countries due, in part, to champions among medical professionals and the power of affected parents. In high-income countries, improved care of the premature baby led to the development of neonatology as a discrete medical sub-specialty and the establishment of neonatal intensive care units [26]. The high prevalence and costs of prematurity have captured the attention of policy-makers and have demanded attention in many high-income countries. In the United States, for example, 11.5 out of every 100 babies born in 2012 were premature. While this rate has declined over recent years, it still represents an increase of more than 22% since 1981 [27]. In addition, the annual societal economic cost in 2005 (medical, educational and lost productivity combined) associated with preterm birth in the United States was at least $26.2 billion. During that same year, the average first-year medical costs, including both inpatient and outpatient care, were about 10 times greater for preterm ($32,325) than for term infants ($3,325). The average length of stay was nine times as long for a preterm newborn (13 days), compared with a baby born at term (1.5 days) [6]. While health plans paid the majority of total allowed costs, out-of-pocket expenses were substantial and significantly higher for premature and low-birthweight newborns, compared with newborns with uncomplicated births [28].

**Figure 4:**
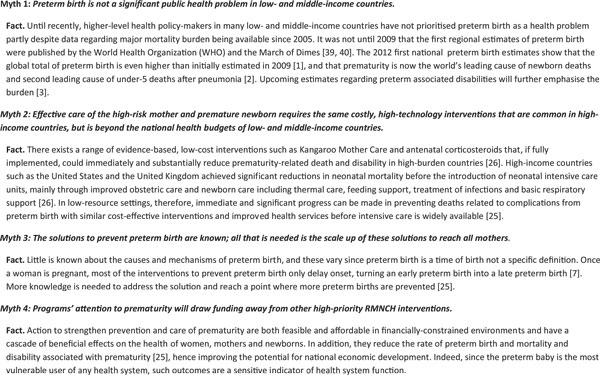
**Myths and misconceptions of preterm birth**. From Born Too Soon report [5].

In low- and middle-income countries, there are common myths and misconceptions that have restricted attention and the implementation of interventions to prevent preterm birth and improve the survival and outcome of premature babies (Figure 4).

## Preterm birth attention, action and research at a tipping point

**Figure 5:**
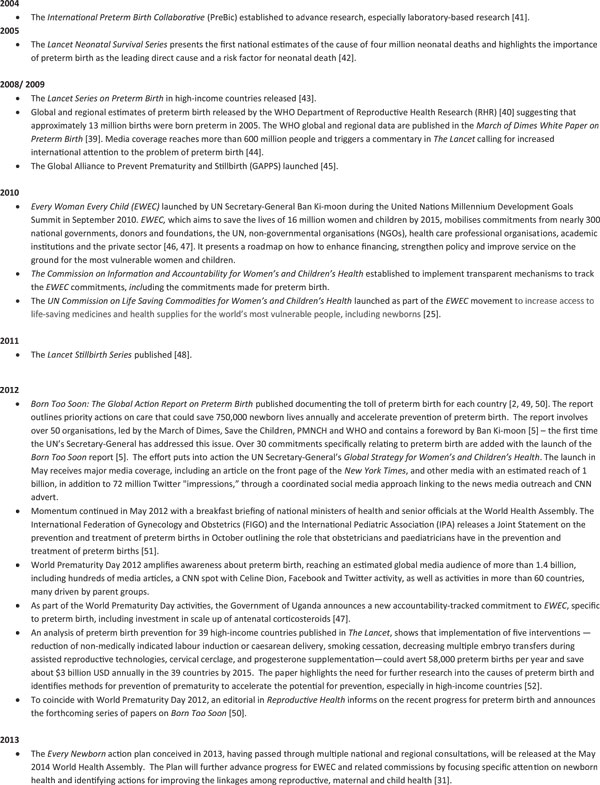
**Milestones in the development of global awareness and action to tackle the problem of newborn survival**.

With the establishment of the MDGs and recent global efforts such as *Every Woman, Every Child *launched by UN Secretary General in support of the *Global Strategy for Women's and Children's Health*, there is growing urgency worldwide to improve health across the RMNCH continuum of care. There is also a growing consensus on what needs to be done, as evidenced by essential packages of interventions for preconception, antenatal and postnatal care [29]. However, despite the large burden, availability of cost-effective solutions and some increase in program funding, a recent global analysis suggests that newborn survival will remain vulnerable on the global agenda without the high-level engagement of policy-makers, adequate funding and specific attention to the problem of preterm birth [30]. Thus, over the past decade, the problem of newborn survival has also begun to receive greater attention globally through an increased volume of publications and meetings. Figure 5 summarises key milestones since 2003 in the movement forward to improve newborn survival.

The recent global mobilisation around the issue of preterm birth complements the growing awareness of newborn health and the importance of quality care at the time of birth to protect the lives of both women and children. As an increasing proportion of under-5 deaths globally, newborns and the importance of their survival have demanded greater action and guidance, especially by country governments. Thus, the group of stakeholders behind *Born Too Soon *came together with countries and other global partners to develop *Every Newborn: an action plan to end preventable deaths *[31]. A roadmap for change, *Every Newborn *will take forward the *Global Strategy for Women's and Children's Health *by identifying actions to improve newborn survival, health and development [31]. Consultation enabling inputs into the action plan is central to the development of *Every Newborn*. Such consultation allows newborn care to be better integrated into RMNCH investments and into programming in countries where specific bottlenecks for high-impact interventions such as essential newborn care, antenatal corticosteroids and Kangaroo Mother Care can be overcome. The first Global Newborn Health Conference held in South Africa in April 2013 provided the launch of these consultations and included several sessions relating to preterm birth prevention and care. The action plan is expected to be released in May 2014 at the WHO World Health Assembly.

### Setting research priorities

Despite the high rate of child death and disability due to prematurity, little is known about how to prevent preterm birth and how best to scale up essential care proven to be practical and affordable. It is, thus, critical to harness recent advances in science and technology and growing global political will to identify novel solutions and to rapidly translate research results into effective global health action. To this end, the Bill & Melinda Gates Foundation, Global Alliance to Prevent Prematurity and Stillbirth, March of Dimes, National Institute of Child Health and Human Development and WHO have convened a series of meetings to advance the visibility of and advocacy and investment in the research required to drive global change in the burden of preterm birth. Several meetings of US-based scientific experts in preterm birth have been held to draft a "Solution Pathway," a comprehensive research agenda to advance preterm birth research across the continuum of discovery, development and delivery science and to improve coordination of research activities. The agenda, a summary of which will be published in an upcoming edition of Lancet Global Health, is expected to result in continued momentum and investment in research. Additionally, WHO and Saving Newborn Lives convened over 90 world experts in neonatal and birth outcomes research to prioritise a list of more than 200 "best ideas" to improve birth outcomes and newborn health by 2025. The ranked priorities will be published soon and include many related to preterm birth [32].

## Preterm birth as a test of the continuum of care

**Figure 6:**
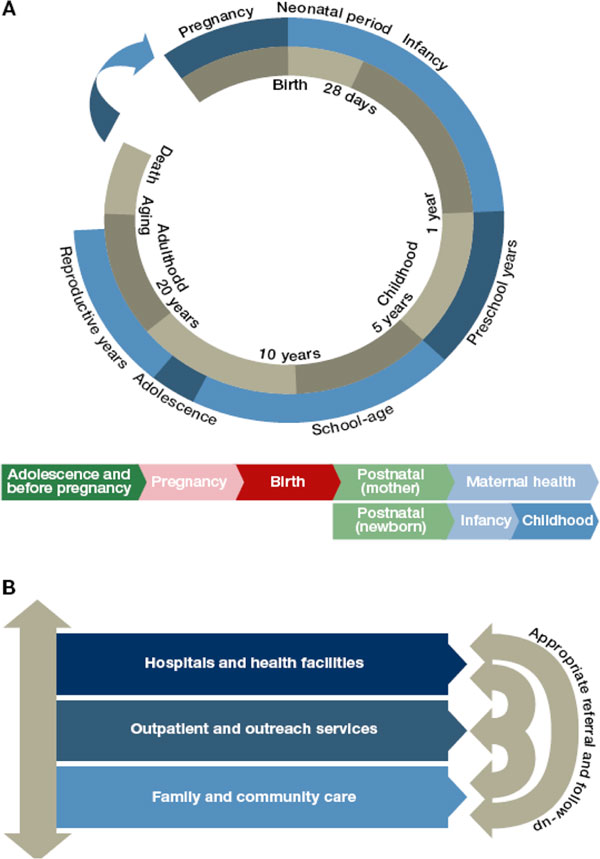
**The Continuum of Care for reproductive, maternal, newborn and child health through the life cycle according to levels of health service delivery**. Source: Born Too Soon report [5], adapted from Kerber et al. 2007 [33].

The *Born Too Soon *supplement in *Reproductive Health *is structured to reflect the continuum of care, a core organizing principle for health systems, which emphasises the delivery of health care packages across time and through service delivery levels. An effective continuum of care addresses the health needs of the adolescent or woman before, during and after her pregnancy, as well as the care of the newborn and child throughout the life cycle, wherever care is provided [33]. Figure 6 shows the continuum of care by time of care giving, throughout the life cycle, from adolescence into pregnancy and birth and then through the neonatal and post-neonatal periods and childhood; and place of caregiving, that is, households, communities and health facilities [33]. Providing RMNCH services through the continuum of care approach has proven cost-effective, and there is evidence that this finding holds for the prevention and treatment of prematurity as well [33-37]. The papers are presented in order of time of caregiving (preconception, during pregnancy and birth and in the postnatal period for care of the preterm baby) [7,26,38]. In each paper the place of caregiving -- at home, at the primary care level and in district and regional hospitals -- is discussed.

While the causes and multiple events during pregnancy that result in a preterm birth require increasingly vigorous research, there are available interventions which, if scaled up and delivered through integrated packages across the RMNCH continuum of care, would have a major and immediate impact on reducing mortality and disability in premature babies. These same interventions would contribute to a modest reduction in preterm birth rates, helping women and their vulnerable babies to survive and thrive.

The supplement emphasizes an action agenda through review of the evidence for these interventions to ensure delivery of the best possible care to all women before, between and during pregnancy; at birth; and to all preterm babies and their mothers and families, wherever they live. In addition, the supplement points to areas of research which require increased attention, funding and collaboration among partners in the governmental, non-governmental and private sectors.

Finally and importantly, the supplement presents actions that require the continued active engagement of all constituencies [25]. Indeed, it is the partners who contributed to the *Born Too Soon *report and the many others who have joined since with their diversity of expertise and experience who represent the strength of this process. Their contributions will help ensure that the actions in this supplement are acted on, sustained and reach the world's poorest families.

## Conflict of interest

The authors declare that they have no competing interests.

## Authors' contributions

The paper was drafted by CPH, MVK and JEL. LM reviewed and contributed.

## Funding

The time of CPH was funded by the March of Dimes and that of JEL and MVK by a grant from Bill & Melinda Gates Foundation to Save the Children's Saving Newborn Lives programme.

The *Born Too Soon *report was funded by March of Dimes, the Partnership for Maternal, Newborn & Child Health and Save the Children. The *Born Too Soon *supplement in Reproductive Health was funded by March of Dimes and Save the Children. Funding from Save the Children is provided by a grant from Bill & Melinda Gates Foundation to Save the Children's Saving Newborn Lives programme.

## List of abbreviations used

MDG: Millennium Development Goal; PMNCH: The Partnership for Maternal, Newborn & Child Health; RMNCH: Reproductive, Maternal, Newborn and Child Health; WHO: World Health Organization.

## Supplementary Material

Additional file 1 **In line with the journal's open peer review policy, copies of the reviewer reports are included as **additional file 1. Click here for file
