# Supplementary material for: Born Toon Soon: Preterm birth matters
Source: Reprod Health. 2013 Nov 15;10(Suppl 1):S1. doi: 10.1186/1742-4755-10-S1-S1 (PMC3828581; doi:10.1186/1742-4755-10-S1-S1)
Supplement: Additional file 1 — In line with the journal's open peer review policy, copies of the reviewer reports are included as additional file 1. [file 1742-4755-10-S1-S1-S1.doc]

**Referee’s comments to the authors– this sheet WILL be seen by the author(s) and published with the article**

| Title | **Preterm birth matters** |
| --- | --- |
| Author(s) | Christopher Howson, Mary Kinney, Lori McDougall and Joy Lawn |
| Referee’s name | Ann Starrs |

**When assessing the work, please consider the following points, where applicable:**

1. Is the question posed by the authors new and well defined?
2. Are the methods appropriate and well described, and are sufficient details provided to replicate the work?
3. Are the data sound and well controlled?
4. Does the manuscript adhere to the relevant standards for reporting and data deposition?
5. Are the discussion and conclusions well balanced and adequately supported by the data?
6. Do the title and abstract accurately convey what has been found?
7. Is the writing acceptable?

Please make your report as constructive and detailed as possible in your comments so that authors have the opportunity to overcome any serious deficiencies that you find and please also divide your comments into the following categories:

- Major Compulsory Revisions (which the author must respond to before a decision on publication can be reached)
- Minor Essential Revisions (such as missing labels on figures, or the wrong use of a term, which the author can be trusted to correct)
- Discretionary Revisions (which are recommendations for improvement but which the author can choose to ignore)

Where possible please supply references to substantiate your comments.

When referring to the manuscript please provide specific page and paragraph citations where appropriate.

**General comments:**

Since the paper is not a research paper, most of the review questions are not fully applicable. Overall, the paper serves as a useful introduction to the topic and to the remaining papers in the series (I assume – I have not seen any of them). However, most of the paper is a review of this history of how prematurity and newborn health have emerged as issues on the global health agenda, detailing the series of publications, events, media attention, etc. that have led to the current level of attention. Since this is an introductory paper, I would encourage the authors to shorten the section “Preterm birth gaining more attention”, or perhaps even shift the key events/developments into a text box, and put a little more in to the final two sections, specifically foreshadowing the key messages/findings from the rest of the papers in the series.

**Major compulsory revisions:** None

**Minor essential revisions:**

There are several typographical errors (e.g., “mobilisng” in the abstract) and sentences that should be broken up (e.g., the first sentence of the full paper), but since the paper is in PDF form and lines are not numbered it is not easy to indicate these suggested changes. If a Word version of the paper could be shared I would be happy to indicate the recommended changes.

There are a few substantive revisions I would strongly encourage, as follows:

p. 3, bottom para, discussion of the increase in NCDs worldwide and the link with prematurity: It is of course significant that 9 million people under the age of 60 die from NCDs, with the greatest burden in Africa; but in terms of the link to maternal health, it would be very helpful to know what is the rate of NCDs, and specifically of diabetes and hypertension, among women of reproductive age.

**Discretionary revisions:**

*(continue on the next sheet)*

*Continued*:

p. 4, 3^rd^ para, 1^st^ sentence: MDG 5 has two targets – target a is the one listed, a ¾ reduction in the MMR; but there is also target 5b, ensuring universal access to reproductive health. It should be referenced as well.

p. 6, 4^th^ para, reference to a meeting in early 2012 which led to progress on the global consensus for preconception care. Was that early 2012, or early 2013? If the authors have been describing events chronologically, as they seem to be doing, I assume this would be 2013. (Note also that the FIGO Congress referenced in this para was held in Nov. 2012).

p. 14, Box 2, Myth 1: Next to last sentence states that prematurity is world’s SECOND leading cause of newborn death, after pneumonia; I think this is supposed to say the second leading cause of CHILD death? See p. 3, 1^st^ paragraph.

**Referee’s comments to the authors– this sheet WILL be seen by the author(s) and published with the article**

| Title | **Paper 1: Preterm Birth Matters** |
| --- | --- |
| Author(s) | Christopher Howson, Mary Kinney, Lori McDougall & Joy Lawn |
| Referee’s name | Joel Segre |

**When assessing the work, please consider the following points, where applicable:**

1. Is the question posed by the authors new and well defined?
2. Are the methods appropriate and well described, and are sufficient details provided to replicate the work?
3. Are the data sound and well controlled?
4. Does the manuscript adhere to the relevant standards for reporting and data deposition?
5. Are the discussion and conclusions well balanced and adequately supported by the data?
6. Do the title and abstract accurately convey what has been found?
7. Is the writing acceptable?

Please make your report as constructive and detailed as possible in your comments so that authors have the opportunity to overcome any serious deficiencies that you find and please also divide your comments into the following categories:

- Major Compulsory Revisions (which the author must respond to before a decision on publication can be reached)
- Minor Essential Revisions (such as missing labels on figures, or the wrong use of a term, which the author can be trusted to correct)
- Discretionary Revisions (which are recommendations for improvement but which the author can choose to ignore)

Where possible please supply references to substantiate your comments.

When referring to the manuscript please provide specific page and paragraph citations where appropriate.

**General comments:** This is a strong paper identifying the magnitude of both the problem and the efforts the team of authors and others have taken to help address PTB. Although I have worked in this space for a few years now, I still learned several new facts from the article. Well done.

**Discretionary revisions:** I have no major revisions to suggest. As far as discretionary revisions go, there may be opportunities to shorten and tighten some sections. There may also be opportunity for slight changes in terminology. For example, the authors refer both to “antenatal steroids” and “antenatal corticosteroids.” The authors also refer to “Saving Newborn Lives” which is a program of “Save the Children.” There is also a subject-verb agreement issue in the abstract where it says “Preterm births is…” It may also be intentional, but the word “series” is not capitalized in the abstract, but it is capitalized on the final page where there are several mentions of the “Series”

Overall, nicely done. I look forward to reading the entire set of articles.

## Comments from the Editor:

For all the articles, these should be seen as independent papers that can be read without the need to read the other papers in the series.
